# Etiological classification and management of dizziness in children: A systematic review and meta-analysis

**DOI:** 10.3389/fneur.2023.1125488

**Published:** 2023-03-02

**Authors:** Jifang Zhang, Qi Zhu, Jiali Shen, Jianyong Chen, Yulian Jin, Qing Zhang, Maoli Duan, Jun Yang

**Affiliations:** ^1^Department of Otorhinolaryngology-Head and Neck Surgery, Xinhua Hospital, Shanghai Jiaotong University School of Medicine, Shanghai, China; ^2^Shanghai Key Laboratory of Translational Medicine on Ear and Nose Diseases, Shanghai, China; ^3^Shanghai Jiaotong University School of Medicine Ear Institute, Shanghai, China; ^4^Department of Otorhinolaryngology-Head and Neck Surgery, Yuyao People's Hospital, Yuyao, Zhejiang, China; ^5^Ear Nose and Throat Patient Area, Trauma and Reparative Medicine Theme, Karolinska University Hospital, Stockholm, Sweden; ^6^Division of Ear, Nose and Throat Diseases, Department of Clinical Science, Intervention and Technology, Karolinska Institutet, Stockholm, Sweden

**Keywords:** dizziness, vestibular disorders, peripheral vertigo, central vertigo, etiology

## Abstract

**Background:**

Dizziness in children, which could not be diagnosed at an early stage in the past, is becoming increasingly clear to a large extent. However, the recognition of the diagnosis and management remains discrepant and controversial due to their complicated and varied etiology. Central and peripheral vestibular disorders, psychogenic and systemic diseases, and genetic pathogeny constitute childhood etiological entities. Further understanding of the etiology and the prevalence of vertigo disorders is of crucial importance and benefit in the diagnosis and management of pediatric patients.

**Methods:**

This systematic review and meta-analysis were conducted by systematically searching Embase, PubMed, the Cochrane Library, CNIK, the Chinese Wan-Fang database, CBM, the Chinese VIP database, and the Web of Science for literature on childhood vertigo disorders published up to May 2022. The literature was evaluated under strict screening and diagnostic criteria. Their quality was assessed using the Agency for Healthcare and Research Quality (AHRQ) standards. The test for homogeneity was conducted to determine the fixed effects model or random-effect model employed.

**Results:**

Twenty-three retrospective cross-sectional studies involving 7,647 children with vertigo disorders were finally included, with an AHRQ score >4 (high or moderate quality). Our results demonstrated that peripheral vertigo (52.20%, 95% CI: 42.9–61.4%) was more common in children than central vertigo (28.7%, 95% CI: 20.8–37.4%), psychogenic vertigo (7.0%, 95% CI: 4.8–10.0%), and other systemic vertigo (4.7%, 95% CI: 2.6–8.2%). The five most common etiological diagnoses associated with peripheral vertigo included benign paroxysmal vertigo of childhood (BPVC) (19.50%, 95% CI: 13.5–28.3%), sinusitis-related diseases (10.7%, 95% CI: −11.2–32.6%), vestibular or semicircular canal dysfunction (9.20%, 95% CI: 5.7–15.0%), benign paroxysmal positional vertigo (BPPV)(7.20%, 95% CI: 3.9–11.5%), and orthostatic dysregulation (6.8%, 95% CI: 3.4–13.0%). Vestibular migraine (20.3%, 95% CI: 15.4–25.2%) was the most seen etiological diagnosis associated with central vertigo in children. In addition, we found the sex-based difference influenced the outcome of psychogenic vertigo and vestibular migraine, while there was no significant difference in other categories of the etiology. For the management of vertigo, symptomatical management is the first choice for most types of vertigo disorder in pediatrics.

**Conclusion:**

Complex etiology and non-specific clinical manifestations of vertigo in pediatrics are challenging for their diagnoses. Reliable diagnosis and effective management depend on the close cooperation of multiple disciplines, combined with comprehensive consideration of the alternative characteristics of vertigo in children with growth and development.

## Introduction

Vertigo and balance problems are not uncommon in children. According to a study on the prevalence of pediatric vertigo and imbalance in the United States, 5.6% of the children with a mean age of 11.5 years had vertigo or imbalance (1), while an epidemiologic study in the United Kingdom found that the prevalence of vertigo in the school-aged population was 5.7% (2). However, vertigo disorders in children are difficult to diagnose and treat effectively since symptoms in children are typically less specific than in adults (3), and results of traditional vestibular tests are not always trustworthy and stable in children (4). Although numerous studies throughout the world have reported on the occurrence and treatment of vertigo problems in children, the diagnosis and classification of etiology remain a point of contention, and comprehensive statistical screening, analysis, and categorization are still needed.

An overview of the prevalent etiologies of vestibular problems in children was published in 2014 (5), in which the inclusion and exclusion criteria were omitted as well as statistical analysis. A systematic review published in 2020 added statistical analysis to the differential diagnosis of vertigo in children (6). Although this review included enough etiologies and provided screening criteria, it lacked heterogeneity analysis of included studies and in-depth exploration of categorization and other etiological contributing factors.

Therefore, the present systematic review and meta-analysis were conducted to investigate the prevalence, categorization, and other contributing aspects of the etiology of pediatric vertigo disorders, to enhance diagnosis and management in this particular population.

## Methods

The Agency for Healthcare and Research Quality (AHRQ) guidelines for retrospective cross-sectional studies were followed in conducting this systematic review and meta-analysis (7).

### Databases and search strategy

**Figure 1:**
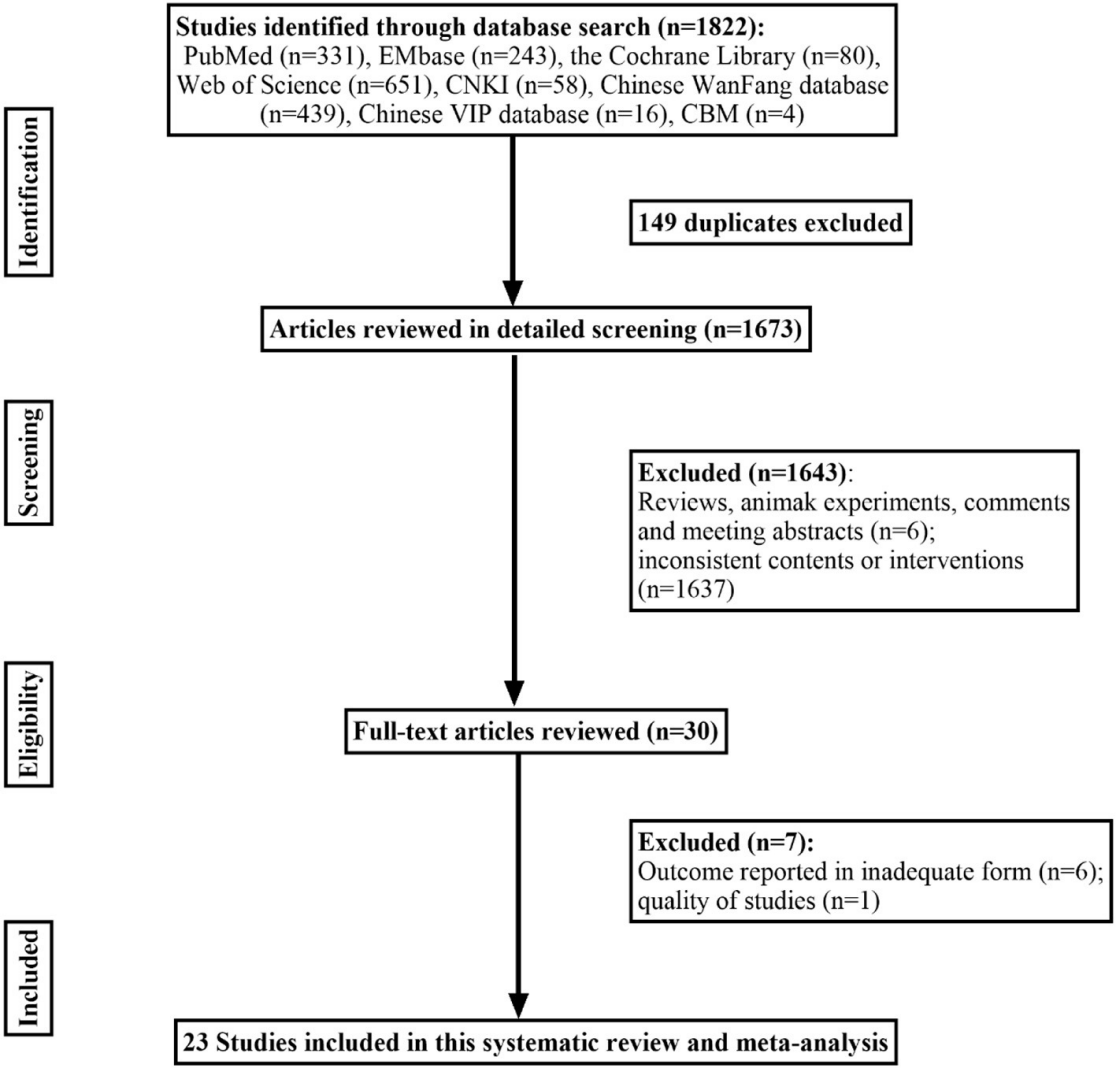
Flow chart of studies included.

PubMed, EMBASE, the Cochrane Library, CNIK, Chinese Wan-Fang database, CBM, Chinese VIP database, and the Web of Science were screened for retrospective studies, cohort studies, case-control studies, or prospective studies on childhood vertigo disorders published up to May 2022. We combined the MeSH terms “Vertigo”, “vestibular diseases”, “Child”, “retrospective study”, “cohort studies”, “case-control studies”, “prospective studies”, and all related entry terms, such as “dizziness”, to obtain sufficient potential articles, and the detailed literature screening process is shown in Figure 1. Each study included was examined in detail to meet with criteria regarding (1) patients who visited a hospital or clinic with vertigo or balance disorder; (2) the detailed medical history and physical examination during diagnostic procedures were definite, such as questionnaire survey, various vestibular tests (coordination tests, Unterberger test, the index-noise test, the heel-tibia test, caloric test, video head impulse test, rotary chair test, vestibular-evoked myo-genic potential), audiological examination or imaging techniques; (3) sufficient data on the prevalence of various etiological causes for vertigo were reported without concealment; (4) original trials. The following studies were excluded: (1) reviews, letters, animal experiments, case reports, and meeting abstracts; (2) patients were excluded for other diseases by subjectivity before inclusion; (3) systematic review and meta-analysis.

### Data extraction and quality assessment

Study characteristics such as author's name, year of publication, sample size, age and sex of participants, country, etiology, and management were collected in the data extraction form. AHRQ standards were used to assess each study included, by which they were then categorized as being of low, moderate, or high quality. The evaluation content is as follows: (1) the source of information (survey, record review) was definite; (2) listing inclusion and exclusion criteria for exposed and unexposed subjects (cases and controls) or referring to previous publications; (3) indicating time period used for identifying patients; (4) indicating whether or not subjects were consecutive if not population-based; (5) indicating if evaluators of subjective components of study were masked to other aspects of the status of the participants; (6) describing any assessments undertaken for quality assurance purposes (e.g., test/retest of primary outcome measurements); (7) explaining any patient exclusions from analysis; (8) describing how confounding was assessed and/or controlled; (9) if applicable, explain how missing data were handled in the analysis; (10) summarizing patient response rates and completeness of data collection; (11) clarifying what follow-up, if any, was expected and the percentage of patients for which incomplete data or follow-up was obtained.

### Statistical analysis

The pooled prevalence of various etiologies of vertigo disorders in children from all eligible studies was calculated by SPSS software. Normality in the distribution of the data collected from all the studies enrolled was checked through normality tests. Then, statistical heterogeneity was assessed through I^2^ and *p*-value by R software (x64 4.1.2). A *p*-value of < 0.1 and the data of I^2^ > 50% indicate high heterogeneity, a random-effects meta-analysis was chosen; a *p*-value of ≥ 0.1 and the data of I^2^ ≥ 50% indicate low heterogeneity, a fixed-effects meta-analysis was then chosen. Besides, subgroup analysis including areas and sexes was also performed. The data of sexes were analyzed by a two-sided Pearson's Chi-square test, and the prevalence of areas was analyzed by a two-tailed Mann–Whitney test.

## Results

A total of 1,822 articles were identified through the database based on our retrieval strategy of which 1,673 were obtained after the removal of duplicate results in EndNote x9. Finally, we obtained 23 retrospective studies in our meta-analysis after screening the titles and abstracts by excluding 1,650 studies of which six were reviews or animal experiments, one was of low study quality and the other 1,643 had inconsistent research content or outcome indicators (8–29) (Figure 1). Our systematic review and meta-analysis involved 7,647 children with vertigo or vestibular imbalance in which 38 types of vertigo etiologies were grouped into five major clades: peripheral vertigo, central vertigo, psychogenic vertigo, other systemic vertigo, and unclassifiable vertigo. All these studies enrolled covered 10 countries, including the United States, Italy, Finland, China, Japan, Turkey, Malaysia, Argentina, Korea, and Mexico. The baseline characteristics of the 23 studies are presented in Table 1.

**Table 1:** Baseline characteristics of included studies.

**References**	**Geographic region**	**Age (Mean ±SD)**	**Sample size**	**Sample size gender (Female/male)**	**Examination**
Bower and Cotton (8)	American	10.6/1.5–19 (Mean/Range)	34	17/17	Symptoms, medical history; physical examinations; audiological examinations; ABR, ENG; CT; metabolic evaluation; platform posturography
D'Agostino et al. (9)	Italy	4–14 (Range)	282	NA	Symptoms, medical history; otoneurologic examination; audiotympanometry; vestibular tests
Riina et al. (10)	Finland	10.9/ ≤ 17 (Mean/Range)	119	63/56	Symptoms; laboratory and otoneurologic tests; imaging studies; neurologic, ophthalmologic, and psychiatric documents
Lijing et al. (11)	China	9.51 ± 2.83	553	212/341	Symptoms; vestibular function tests; audiological examinations; imaging studies
O'Reilly et al. (12)	American	≤ 18 (Range)	2546	NA	NA
Ueda et al. (13)	Japan	4-15 (Range)	40	17/23	Vestibular and balance tests; symptoms; neurological, otorhinolaryngological examinations
O'Reilly et al. (14)	American	9.7 ± 5	132	63/69	Neurological and physical documents; audiometry; vestibular and balance tests; laboratory tests; imaging examinations
Erdogan et al. (15)	Turkey	11.5 ± 4.1	30	13/17	Symptoms; laboratory tests; EEG and magnetic resonance imaging
Salim et al. (18)	Malaysia	5–18 (Range)	24	12/12	Symptoms; neurological examinations; pure audiometry and tympanometry; laboratory tests; brain imaging examinations
Goto et al. (17)	Japan	8.7 ± 3	77	35/42	Ophthalmology examination; vestibular and balance tests; BHIT; pure audiometry; ENG, ABR, VEMP
Batu et al. (16)	Turkey	7.5/ ≤ 18 (Mean/Range)	100	54/46	Symptoms; physical, neurological and audiological fingings; imaging examinations; EEG
Sommerfleck et al. (19)	Argentina	10/1–18 (Median/Range)	206	99/107	Full anamnesis; physical and otoneurological examinations
Lee et al. (20)	South Korea	12.9/ ≤ 18 (Mean/Range)	411	230/181	Detailed medical history; physical examinations; positional and positioning maneuvers; caloric tests; pursuit, saccadic, and optokinetic tests; VEMP, ABR, HIT; pure tone audiometry, speech audiometry, and impedance audiometry tests; EEG, ECHO, and imaging examinations
Zeng et al. (21)	China	9.0/ ≤ 18 (Median/Range)	82	46/36	Pure tone audiometry, audiological examination; ABR, OAE, VEMP; vestibular function examinations; saccadic, and optokinetic tests; positional and positioning maneuvers; DHI; imaging examinations; multidisciplinary consultation
Li et al. (22)	China	10 ± 3.91	46	24/22	Symptoms, medical history; optokinetic tests, positional tests, Rmberg tests and Fukuda tests, HIT; pure tone audiometry; vestibular function examinations; imaging examinations
Li et al. (23)	China	9.33 ± 2.84	54	33/21	Symptoms; vestibular function tests;
Yilmaz and Abseyi (24)	Turkey	13.9/12–19 (Mean/Range)	301	205/96	Symptoms, medical history; cardiological, neurological, ophtalmologic, audiological, psychiatric examinations; imaging examination; EEG
Karatoprak et al. (26)	Turkey	11.7 ± 4.1	59	36/23	Symptoms, medical history; physical, neurological, otological, ophthalmological, cardiovascular, and psychiatric examinations; laboratory examinations; cranial MRI; EEG
Ishiwara-Niembro et al. (25)	Mexico	14.5 ± 3.9	212	107/105	Symptoms, medical history; audiological, physical, neurological, and otological examinations
Wang et al. (27)	American	12.5 ± 4.9	1,021	624/397	Symptoms; otological and neurological examinations; vestibular testing; positional maneuver
Grasso et al. (28)	Italy	1–18 (Range)	757	420/337	Symptoms; neurological, otorhinolaryngological examinations; imaging examinations

CT, Computed tomographic scans; BHIT, Bed side head impulse test; ENG, Electronystamography; ABR, Auditory brainstem response; VEMP, Vestibular myogenic potentials; EEG, Electroencephalography; ECHO, Echocardiography; OAE, Otoacoustic emissions; DHI, Dizziness handicap index; HIT, Head impulse test; MRI, Magnetic resonance imaging; NA, not available.

The quality of all included studies was assessed according to the AHRQ standards and the scores of each study are presented in Table 2, respectively. Of 23 studies, 17 were rated as high quality with scores ≥ 8, and the others were rated as moderate quality with scores ranging between 4 and 7.

**Table 2:** Quality assessment of retrospective studies.

**References**	**(1)**	**(2)**	**(3)**	**(4)**	**(5)**	**(6)**	**(7)**	**(8)**	**(9)**	**(10)**	**(11)**	**Score**	**Quality**
Bower and Cotton (8)	1	0	1	1	1	1	0	1	1	1	0	8	High
D'Agostino et al. (9)	1	0	1	1	1	1	0	1	1	1	0	8	High
Riina et al. (10)	1	0	1	1	1	1	0	1	1	1	0	8	High
Lijing et al. (11)	1	0	1	1	1	1	1	1	1	1	0	9	High
O'Reilly et al. (12)	0	0	1	1	1	0	0	0	1	1	0	5	Moderate
Ueda et al. (13)	1	0	1	1	1	1	0	1	1	1	0	8	High
O'Reilly et al. (14)	1	0	1	1	1	1	0	1	1	0	0	7	Moderate
Erdogan et al. (15)	1	0	1	1	1	1	0	1	1	1	1	9	High
Salim et al. (18)	1	0	1	1	1	0	0	1	1	0	0	6	Moderate
Goto et al. (17)	1	0	1	1	1	1	1	1	1	1	0	9	High
Batu et al. (16)	1	0	1	1	1	1	0	1	1	1	0	8	High
Sommerfleck et al. (19)	1	0	1	1	1	1	0	1	1	1	0	8	High
Lee et al. (20)	1	0	1	1	1	1	0	1	1	1	0	8	High
Zeng et al. (21)	1	0	1	1	1	1	1	1	1	1	1	10	High
Li et al. (22)	1	0	1	1	1	1	0	0	1	1	0	7	Moderate
Li et al. (23)	1	0	1	1	1	0	1	1	1	1	1	9	High
Yilmaz and Abseyi (24)	1	0	1	1	1	1	0	1	1	1	0	8	High
Karatoprak et al. (26)	1	0	1	1	1	1	1	1	1	1	1	10	High
Ishiwara-Niembro et al. (25)	1	0	1	1	1	0	0	1	1	1	0	7	Moderate
Wang et al. (27)	1	0	1	1	1	1	1	1	1	1	0	9	High
Grasso et al. (28)	1	0	1	1	1	0	0	1	1	1	0	7	Moderate

(1) The source of information (survey, record review) was definite; (2) listing inclusion and exclusion criteria for exposed and unexposed subjects (cases and controls) or referring to previous publications; (3) indicating time period used for identifying patients; (4) indicating whether or not subjects were consecutive if not population-based; (5) indicating if evaluators of subjective components of study were masked to other aspects of the status of the participants; (6) describing any assessments undertaken for quality assurance purposes (e.g., test/retest of primary outcome measurements); (7) explaining any patient exclusion from analysis; (8) describing how confounding was assessed and/or controlled; (9) if applicable, explain how missing data were handled in the analysis; (10) summarizing patient response rates and completeness of data collection; (11) clarifying what follow-up, if any, was expected and the percentage of patients for which incomplete data or follow-up was obtained.

The prevalence of etiology collected and 95% CI in pediatric patients were calculated for each study, respectively, with heterogeneity. A total of 37 kinds of etiological diagnoses were categorized as peripheral vertigo disorders, central vertigo disorders, psychogenic vertigo, or other systemic diseases (30, 31). In addition, some vertigo symptoms or balance problems that could not be diagnosed accurately were categorized as unclassified vertigo and excluded from data analysis. A conclusion can be made from these results that peripheral vertigo (52.20%, 95% CI: 42.9–61.4%) was more common in children than central vertigo (28.70%, 95% CI: 20.8–37.4%), psychogenic vertigo (7.0%, 95% CI: 4.8–10.0%), and other systemic vertigo (4.70%, 95% CI: 2.6–8.2%).

### Peripheral vertigo disorders

**Figure 2:**
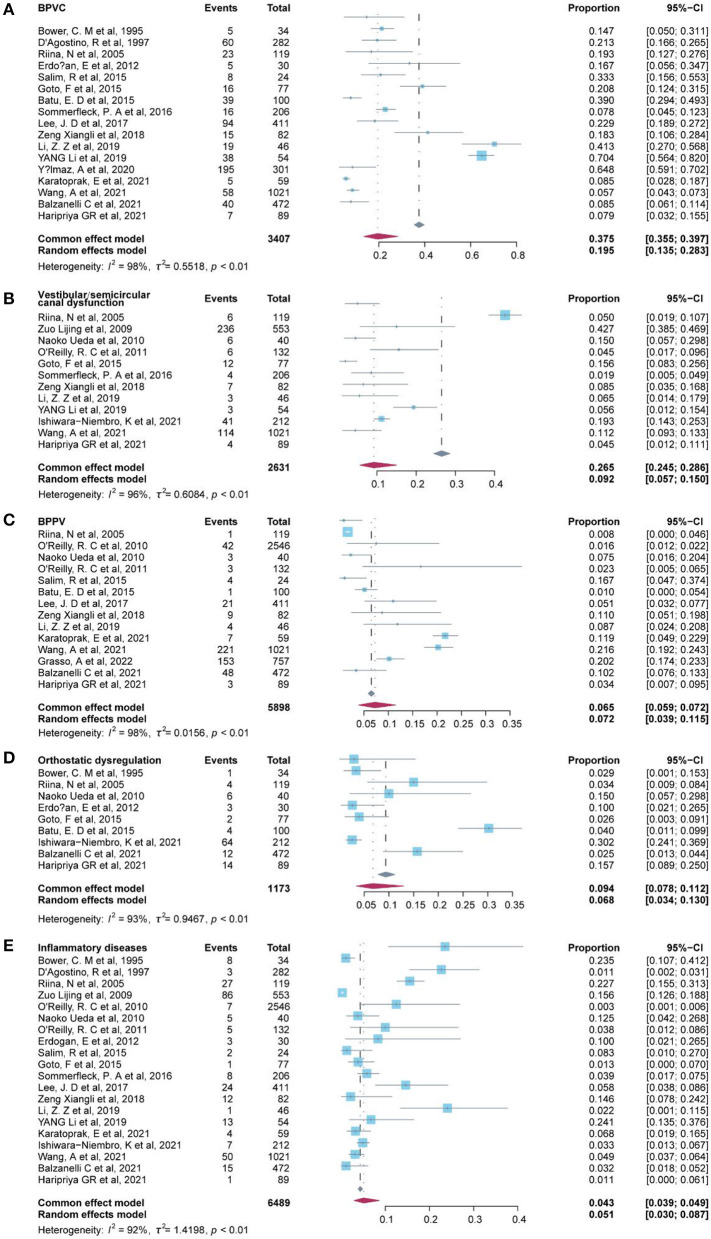
Forest plot of common diagnosis in peripheral vertigo.

A total of 17 of the etiological diagnosis classified as peripheral vertigo disorders were reported in 23 studies (Table 3), and the six most common etiological diagnoses were benign paroxysmal vertigo of childhood (BPVC) (19.50%, 95% CI: 13.5–28.3%), sinusitis-related diseases (10.7%, 95% CI: −11.2–32.6%), vestibular or semicircular canal dysfunction (9.20%, 95% CI: 5.7–15.0%), benign paroxysmal positional vertigo (BPPV) (7.2%, 95% CI: 3.9–11.5%), orthostatic dysregulation (6.8%, 95% CI: 3.4%-13.0%), and inflammatory diseases (5.10%, 95% CI: 3.0–8.7%) (Figure 2). Notably, the sinusitis-related disease was reported in only two studies, in which the data were indicated as low representative. Inflammatory diseases included otitis media with effusion and parotitis, and vestibular neuritis was included in peripheral vascular/neuropathy where the mechanism of vestibular neuritis lies in vestibular neuropathy, which is a type of peripheral neuritis. Labyrinthitis was included in labyrinthine disorders. A fixed-effect model was applied to pool these studies of cholesteatoma, myringosclerosis or ear wax, meningitis-associated diseases, and cochlear implantation since substantial heterogeneity was low (I^2^ = 0). A random-effects model was used in the other diagnosis of peripheral vertigo disorders since the heterogeneity of these etiologies was high with I^2^ ≥ 50% or a *p*-value of < 0.1.

**Table 3:** Pooled analysis of each etiology for peripheral vertigo disorders.

**Etiology**	**Number of included studies**	**Pooled effects**		**Heterogeneity**		**Analysis model**
		**Rate Estimate (%)**	**95% CI (%)**	***I* ^2^**	***P*-value**	
BPV	15	21.90%	14.9–32.1%	98%	< 0.01	Random-effected model
Sinusitis-related	2	10.70%	−11.2–32.6%	88.10%	0.004	Random-effected model
Vestibular/semicircular canal dysfunction	11	9.80%	5.9–16.2%	97%	< 0.01	Random-effected model
Orthostatic dysregulation	7	7.50%	3.5–16.2%	89%	< 0.01	Random-effected model
BPPV	12	7.40%	3.6–12.3%	98%	< 0.01	Random-effected model
Inflammatory diseases	18	7.10%	3.9–11%	96%	< 0.01	Random-effected model
Malformation (EVA included)	4	4.50%	1.3–9.2%	80%	< 0.01	Random-effected model
Peripheral vascular/neuropathy	6	3.70%	1.1–12.4%	91%	< 0.01	Random-effected model
Sudden deafness	7	3.10%	1.6–6.1%	49%	0.07	Random-effected model
labyrinthine disorder	9	3%	1.4–6.3%	95%	< 0.01	Random-effected model
Meniere's disease	11	2.40%	0.3–4.4%	89%	< 0.01	Random-effected model
Cholesteatoma	3	2.20%	0.1–4.3%	0	0.58	Fixed-effect model
Lymphedema/ Perilymphatic fistula	3	2.10%	0.3–5.8%	63%	0.07	Random-effected model
Myringosclerosis /Ear Wax	3	0.80%	0.0–2.3%	0	0.47	Fixed-effect model
Meningitis associated	2	0.90%	0.2–1.6%	0	1	Fixed-effect model
Ototoixc drugs	2	3.80%	−2.4–9.9%	93.80%	0	Random-effected model
Cochlear implantation	2	0.70%	−0.2–1.6%	0	0.39	Fixed-effect model
Total	21	53.90%	41.9–65.6%	99%	0	Random-effected model

BPVC, Benign paroxysmal vertigo of childhood; BPPV, Benign paroxysmal positional vertigo; EVA, Enlarged Vestibular Aqueduct.

### Central vertigo disorders

**Figure 3:**
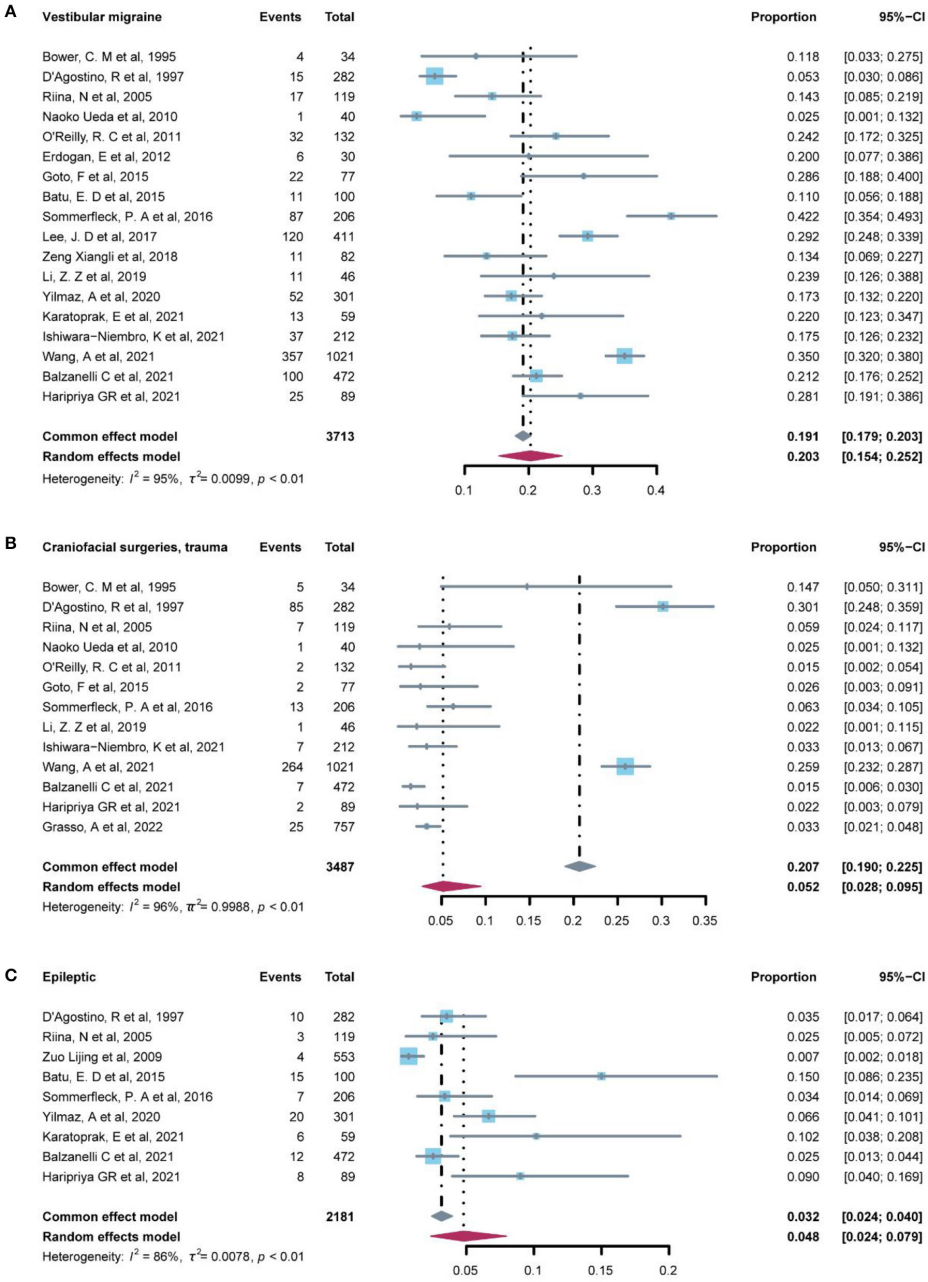
Forest plot of common diagnosis in central vertigo.

The prevalence of central vertigo disorders was evaluated in 19 studies (Table 4) which included 10 etiological diagnoses. The most common diagnosis was vestibular migraine (20.3%, 95% CI: 15.4–25.2%), which was followed by craniofacial surgeries/ trauma (5.20%, 95% CI: 2.8–9.5%), and epileptic (4.80%, 95% CI: 2.4–7.9%) (Figure 3). Associated diagnoses that occurred only once in all studies enrolled were lightning, and motor or developmental delay. The fixed-effect model was used to pool the results in cervical vertigo and movement disorders with I^2^ < 50% (low heterogeneity) and the rest diagnoses in central vertigo disorders were assessed in the random-effect model.

**Table 4:** Pooled analysis of each etiology for central vertigo.

**Etiology**	**Number of included studies**	**Pooled effects**		**Heterogeneity**		**Analysis model**
		**Rate Estimate (%)**	**95% CI (%)**	***I* ^2^**	***P*-value**	
Migraine associated	16	19.10%	13.9–24.9%	94%	< 0.01	Random-effect model
Craniofacial surgries/tauma	11	6.30%	3.3–11.9%	95%	< 0.01	Random-effect model
Epileptic	7	4.90%	2.1–8.7%	88%	< 0.01	Random-effect model
Cervical vertigo	3	2.60%	0.2–5.1%	0	0.7	Fixed-effect model
Brain injury/malformation/ tumor	5	1.50%	0.4–5.5%	87%	< 0.01	Random-effect model
Movement disorder	4	1.40%	0.7–2.3%	17%	0.31	Fixed-effect model
Central nervous system disorders/ Encephalopathy	12	0.50%	0.29–7.6%	87%	< 0.01	Random-effect model
Vestibulotoxic drugs	1	NA	NA	NA	NA	NA
Lightning	1	NA	NA	NA	NA	NA
Motor/developmental delay	1	NA	NA	NA	NA	NA
Total	19	28.40%	19.8–37.8%	99%	0	Random-effect model

NA, Not available.

### Psychogenic vertigo disorders

**Figure 4:**
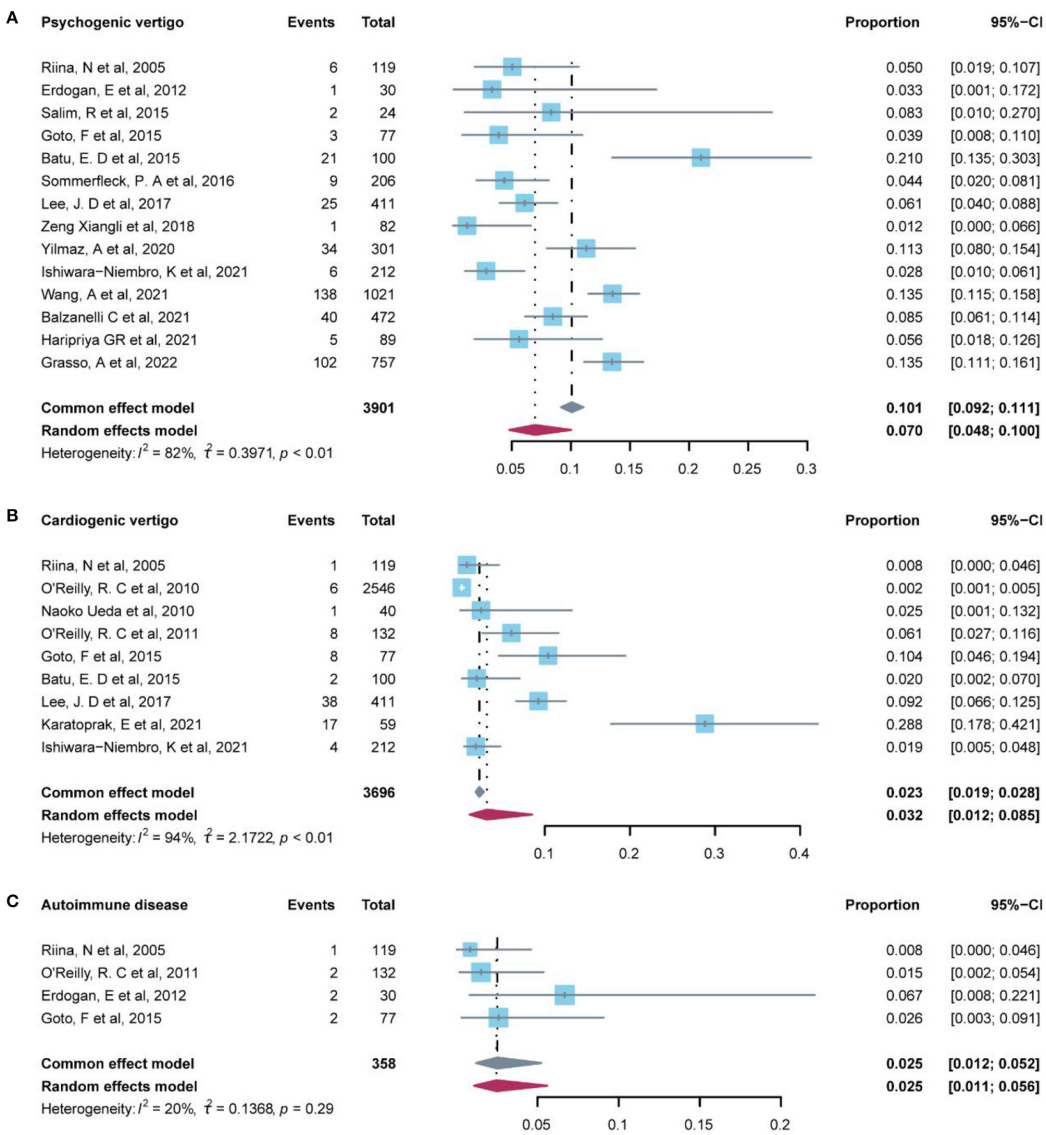
Forest plot of psychogenic vertigo and common diagnosis in other systematic disease.

A total of 12 studies reported the prevalence of psychogenic vertigo (Table 5). Our pooled evidence suggested that psychogenic vertigo disorder (7.00%, 95% CI: 4.8–10.0%) was more common than other systemic diseases (4.70%, 95% CI: 2.6–8.2%), significant heterogeneity was detected among these studies (I^2^ = 82%, *P*-value < 0.01), and the random-effect model was conducted (Figure 4).

**Table 5:** Pooled analysis of each etiology for Psychogenic vertigo and other systematic disorders.

**Etiology**	**Number of included studies**	**Pooled effects**		**Heterogeneity**		**Analysis model**

		**Rate Estimate** **(%)**	**95% CI (%)**	*I* ^2^	***P*** **-value**	
Psychogenic vertigo	12	7.40%	4.9–11.2%	83%	< 0.01	Random-effect model
Infectious disease	2	9.00%	−6.7–24.6%	98.90%	0	Random-effect model
Cardiogenic vertigo	9	3.50%	1.3–9.4%	94%	< 0.01	Random-effect model
Autoimmune disease	4	2.50%	1.1–5.6%	20%	0.29	fixed-effect model
Oculomotor abnormality	2	1.00%	−0.3–2.3%	0	1	fixed-effect model
Ophthalmic disease	8	0.80%	0.1–1.9%	74%	< 0.01	Random-effect model
Iron deficiency anemia	1	NA	NA	NA	NA	NA
Pendred Syndrome	1	NA	NA	NA	NA	NA
Syncope	1	NA	NA	NA	NA	NA
Cytomegalovirus	1	NA	NA	NA	NA	NA
Total (other systematic diseases)	15	4.60%	2.4–8.9%	94%	0	Random-effect model

NA, Not available.

### Other systemic diseases

Other systemic diseases included in 15 studies in Table 5 indicated that infectious disease (9.00%, 95% CI: −6.7–24.6%) and cardiogenic vertigo disease (3.5%, 95% CI: 1.3–9.4%) were relatively common in this group, followed by autoimmune disease (2.50%, 95% CI: 1.1–5.6%) (Figure 4). Infectious disease was reported in only two studies which indicated that the data were low representative. The fixed-effect model was used in autoimmune diseases (I^2^ = 20%, *P*-value = 1) and oculomotor abnormality (I^2^ = 0, *P*-value = 1). Iron deficiency anemia, Pendred syndrome, syncope, and cytomegalovirus were, respectively, reported by only one study.

### The factor of gender and country/area

**Figure 5:**
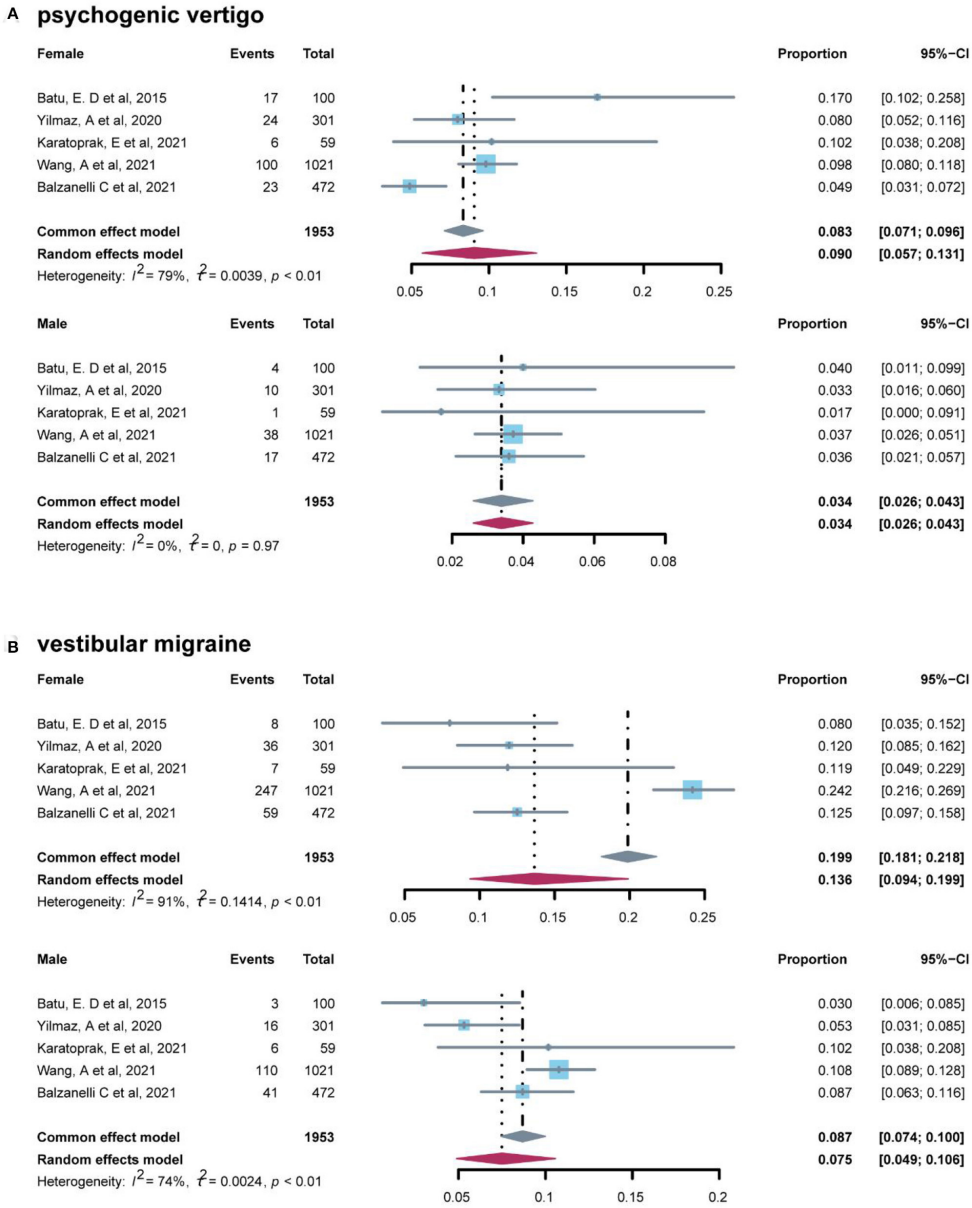
Forest plot of psychogenic vertigo and vestibular migraine in female and male.

To further explore the impact of other factors on the prevalence of vertigo disorders, we performed a two-sided Chi-square (χ^2^) test for sex (Table 6) and a two-tailed Mann–Whitney test for the region (Table 7). Data on sex were extracted from studies which reported samples from different sexes, and three of etiological diagnoses were included. The *p*-values of psychogenic vertigo disease and vestibular migraine were 0.001 and 0.002, respectively, which indicated sex difference was statistically significant (*P* < 0.05) in these two diagnoses. Forest plots of studies for men and women in the diagnosis are presented in Figure 5, the results of which indicated that women (10.3%, 95% CI: 7.3–13.6%) had a higher prevalence rate in psychogenic vertigo disease than men (3.3%, 95% CI: 2.4–4.4%).

**Table 6:** The prevalence of BPVC, vestibular migraine and psychogenic vertigo in different gender.

	**Total sample**	**Sex**, ***n*** **(%)**		**Statistics**	
		**Girls**	**Boys**	**F for χ^2^**	*P*-value^*^
BPVC	n=297	188 (63.3%)	109 (36.7%)	1.0000	0.4170
Vestibular migraine	n=433	298 (68.8%)	135 (31.2%)	1.0000	0.0309
Psychogenic vertigo	n=200	147 (73.5%)	53 (26.5%)	1.0000	0.0046

χ^2^, Pearson's chi-square test; ^*^two-sided t-test.

**Table 7:** The prevalence of common diagnosis in different area.

	**Studies included**	**Sample (case/total)**		**Statistics**	
		**Asian regions**	**European and American regions**	**F for χ^2^**	*P*-value^*^
Peripheral vertigo disorders (Total)	21	1,191/1,777	1,280/5,309	0.4783	0.5318
BPVC	15	434/1,184	162/1,662	0.1779	0.7028
Vestibular/semicircular canal dysfunction	11	264/798	171/1,690	0.2530	0.8295
Orthostatic dysregulation	7	11/147	69/365	433.0286	0.3138
BPPV	12	38/657	420/4,575	215.2444	0.0615
Central vertigo disorders (total)	19	227/1,363	1242/5,309	28.2191	0.0553
Vestibular migraine	16	182/786	549/2,006	11.1188	0.2650
Craniofacial surgries/ trauma.	11	4/163	408/2,763	24443.1429	0.3808
Epileptic	7	39/954	20/607	0.2167	0.3812

^*^Mann–Whitney test, two-tailed.

**Figure 6:**
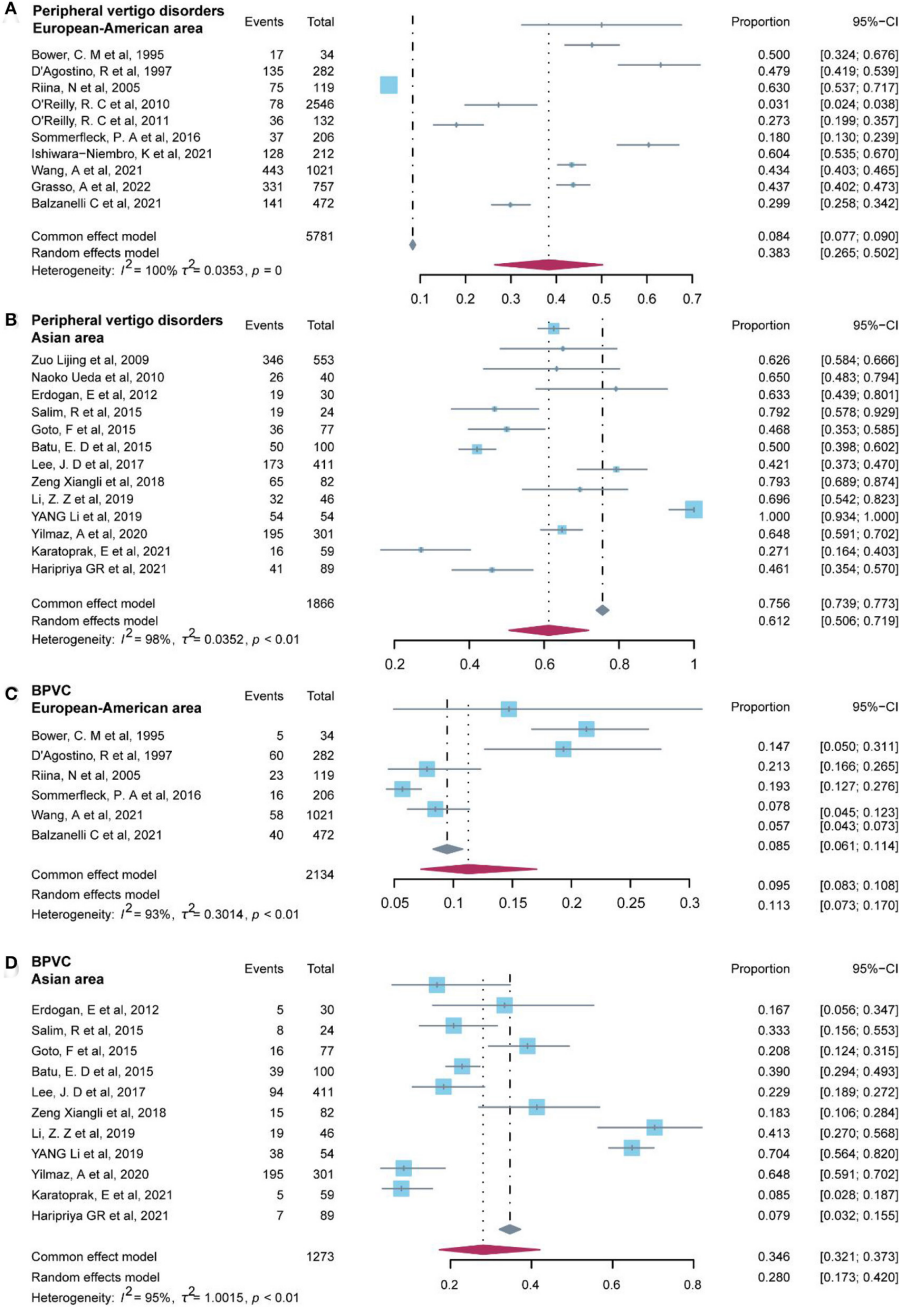
Forest plot of peripheral vertigo disorders and BPVC in different areas.

Additionally, we examined the non-parametric test (two-tailed Mann–Whitney test) on the prevalence of common diagnoses in peripheral vertigo disorders and central vertigo disorders for regions categorized as Asian area and European–American areas. Studies included in the Asian area were from China, Japan, Turkey, Malaysia, India, and South Korea, on the other hand, studies conducted in Italy, Finland, the United States, Argentina, and Mexico were included in the European–American area. The results of the *p*-value in the statistical analysis indicated the difference was significant (*P* < 0.05) in peripheral vertigo disorders (*P* = 0.003), and BPVC (*P* = 0.038). Then, we used the R software to test for regional heterogeneity. As a result of the high heterogeneity, the random model we employed revealed that the Asia area (61.2%, 95% CI: 50.6–71.9%) had a greater prevalence of peripheral vertigo disorders than the European–American area (38.3%, 95% CI: 26.5–50.2%), and BPVC revealed the same results (Asian area: 28.0%, 95% CI: 17.3–42.0%; European–American area: 11.3%, 95% CI: 7.3–17.0%) (Figure 6). While the other common diagnoses did not show the difference between the two regions (*P* ≥ 0.05).

## Management

Management and prognosis of 13 etiological diagnoses in studies enrolled were given in Table 8. BPVC, migraine, otitis media, and vestibular neuritis were reported in more than one study. The remaining etiologies mentioned were BPPV, sinusitis-related diseases, postural hypotension, vitamin B12 deficiency, myringosclerosis, psychogenic vertigo, inner ear malformations, Meniere's disease, and enlarged vestibular aqueduct (EVA). Symptoms of diseases reported in studies met the essential diagnostic criteria published but some children reported atypical symptoms or multifactorial symptoms, which resulted in different management schedules in some studies. The most common management of vertigo disorders in children was symptomatic therapy.

**Table 8:** Information of management and prognosis in studies included.

**References**	**Symptom**	**Management**	**Prognosis**
Erdogan et al. (15)	Headache, blurred vision, tinnitus	Diphenhydramine	Clinical recovery
Salim et al. (18)	Spinning vertigo, nystagmus, nausea and vomiting with motion, light and sound sensitive, and relieved by sleeping.	Anti-migraine medication	NA
Goto et al. (17)	Episodic vertigo for few minutes	Pharmaceutical therapy/non-pharmacological rehabilitation treatment	Improvement
Li et al. (23)	Headache, photophobia, dizziness	Symptomatic therapy (betahistine mesylate),neurotrophic drugs	Clinical improvement
Erdogan et al. (15)	Recurrent headaches with light/sounds sensitive, blurred vision, loss of balance	Propranolol treatment	Clinical improvement
Goto et al. (17)	Recurrent headaches with light/sounds sensitive, blurred vision, loss of balance	Valproic acid, β-blocker/non-pharmacological rehabilitation treatment	Improvement
Erdogan et al. (15)	Tinnitus, nausea and vomiting	Antibiotic therapy	Clinical recovery
Li et al. (23)	Recurrent headaches, gait instability	Surgery, antibiotic therapy	Clinical improvement
Li et al. (23)	Persistent vertigo without headache	Surgery, antibiotic therapy	Clinical improvement
Erdogan et al. (15)	Nausea and vomiting, headache	Antihistamines and antiemetic therapy	Clinical improvement
Salim et al. (18)	Symptoms were unspecific in children	Modified Epley canalith repositioning procedure	Clinical recovery
Erdogan et al. (15)	Headache,dizziness and loss of balance, nausea, and vomiting	Antibiotic therapy	Clinical improvement
Erdogan et al. (15)	Amaurosis, loss of balance	Specific recommendations and assistance	NA
Erdogan et al. (15)	Loss of balance, blurred vision	Vitamin B12 supplement	Clinical improvement
Erdogan et al. (15)	Headahe, loss of balance, nausea, and vomiting	Preventive treatment and clinical follow-up	Improvement
Erdogan et al. (15)	Anxiety,blurred vision	Psychiatric treatments	Clinical improvement
Zeng et al. (21)	Hearing impairment,loss of balance	Family custody	Improvement
Zeng et al. (21)	Loss of balance, nausea, and vomiting	Conventional therapy, triggers avoidance	Improvement
Li et al. (23)	Recurrent vertigo without hearing loss; recurrent headaches with light/sounds sensitive	Flunarizine and Betahistine symptomatic therapy	Clinical improvement

EVA, Enlarged vestibular aqueduct.

## Discussion

Studies on pediatric vestibular disorders have grown in recent years with an increase in attention toward vertigo disorder in children. This systematic review and meta-analysis were conducted to analyze various etiologies related to vertigo and imbalance disorders in children and the management schedule in statistical methods. Reports of diagnosis classification in vertigo disorders might be different in various studies. Tusa et al. (32) reported the common causes of dizziness in children and grouped them into acute vertigo, spells of dizziness, or chronic dizziness. However, Devaraja (4) and Gioacchini et al. (5) gave reviews of the causes of vertigo in children without classification. A systematic review (6) categorized differential diagnoses of vertigo in children according to occurrence percentage. Jahn (33) reported that the causes were classified as peripheral vestibular disorders, central vestibular disorders, and non-vestibular dizziness. We separate psychogenic vertigo and other systemic vertigo into two individual groups apart from peripheral vertigo and central vertigo, since more evidence was provided that mental problems and systemic disease interacted closely with vestibular disorders in children (31, 34).

Our results suggested that vestibular migraine had the most occurrence, followed by peripheral vestibular disorders, including BPVC, sinusitis-related diseases, vestibular or semicircular canal dysfunction, BPPV, orthostatic dysregulation, which had a higher occurrence rate than other causes. Besides, children's psychological aspects are crucial in the development of vertigo. Notably, studies that involved sinusitis-related disease and infectious disease were inadequate, although the occurrence rate was relatively high. Additionally, it has been disputed whether peripheral vertigo disorders or vestibular vertigo disorders caused BPVC because the mechanism of BPVC is still poorly understood. In 1977, Eviatar (35) included BPVC in the category of peripheral vertigo, but Karatas (36) considered it to be central vertigo in 2008. Moreover, according to the Bárány Society and the International Headache Society in 2021 (37), some pediatric patients with vertigo fit both BPVC criteria as well as VM criteria and introduced a new term “Recurrent Vertigo of Childhood” (RVC) to replace BPVC, which led to heterogeneity to some degree. In a systematic review, vestibular migraine also indicated a higher occurrence rate than BPVC (6) though this review differed in databases searched and methodology. There was a multi-central study in which BPVC was the most common cause in preschool and school-age children (up to and including 12-year-old), while vestibular migraine was the most common in adolescents (13- to 18-year-old). Additionally, Wang et al. (27) reported that 44.4% of patients received multiple contributing diagnoses and vestibular migraine was diagnosed frequently compared with other diseases. Therefore, we consider the difference in occurrence between BPVC, and vestibular migraine resulting from the age of the population included or the interaction of multiple vestibular disorders.

Previously, few studies have focused on the impact of gender on the causes of vertigo disorder in children, while the age group had more concerns. Evidence obtained indicated that girls had a higher risk of having vertigo than boys (24, 35), and gender was identified as an independent risk factor in adolescents (36). Our study reports that girls are more likely to experience psychogenic vertigo disease and vestibular migraine, which is mainly explained by the influence of estrogen and the menstrual cycle in adolescent girls. Gender specificity in other etiological diagnoses was scant in the studies included, more detailed studies are needed. Additionally, there were regional variations in the occurrence of peripheral vertigo disorders, particularly BPVC. Further research is necessary in light of this finding on the greater prevalence of peripheral vertigo disorders in Asian youngsters. Given the atypical presentation of vertigo, inability to explain symptoms, and drug toxicity in children, the management and prognosis of children are very different from adults. Our study indicates that most patients recovered or improved after symptomatic treatment, and non-pharmacological rehabilitation treatment was reported to be effective in some cases (17) .

## Limitations

There is insufficient literature available in some etiologies for statistical analysis and data consolidation, such as cochlear implantation, otolithic dysfunction, sinusitis-related disease, lightning, and developmental delay. Besides, all the studies included stated that the symptoms of vertigo disorders had met the diagnostic criteria; however, the number of unclassified vertigoes was notable. Conventional examinations, such as vestibular tests and clinical tests, are not uniformly reliable in children, and protocols or standards suitable for them remain lacking.

## Conclusion

In summary, this systematic review and meta-analysis identified the common etiology of vertigo, gender specificity in vestibular migraine and psychogenic vertigo disease, and the common management schedule in the children. The pooled results and review in the statistical analysis could provide the probability of diagnosis for the physician. Further studies are needed to explore more impactive factors which are helpful to improve the accuracy of diagnosis.

## Data availability statement

The original contributions presented in the study are included in the article/supplementary material, further inquiries can be directed to the corresponding authors.

## Author contributions

JZ: study conception, data quality control, and wrote the manuscript. QZhu: study conception, drafted the manuscript, and graphic abstract. JS: study conception and drafted the manuscript. JC: study conception. YJ and QZha: data quality control. MD and JY: data quality control and wrote the manuscript. All authors contributed to the article and approved the submitted version.
